# Sexual Disability in Low Back Pain: Diagnostic and Therapeutic Framework for Physical Therapists

**DOI:** 10.3390/healthcare12010080

**Published:** 2023-12-29

**Authors:** Carla Vanti, Silvano Ferrari, Marco Chiodini, Cesare Olivoni, Arianna Bortolami, Paolo Pillastrini

**Affiliations:** 1 Department of Biomedical and Neuromotor Sciences (DIBINEM), Alma Mater University of Bologna, Via Massarenti 9, 40138 Bologna, Italy; carla.vanti@unibo.it (C.V.); paolo.pillastrini@unibo.it (P.P.); 2 Studio Arcobaleno, Via Ramazzini, 7, 20129 Milano, Italy; silvano.ferrari@fastwebnet.it; 3 Poliambulatorio Medico Associato, Via Monsignor Bertazzoni, 1, 46027 San Benedetto Po, Italy; chiodini_m@hotmail.it; 4 Studio Associato Fisioterapico Gallinucci Olivoni, Piazzale Marconi, 3/4, 48025 Riolo Terme, Italy; cesare.olivoni@gmail.com; 5 Studio Pelvic Floor, Via Gattamelata, 134/C, 35128 Padova, Italy; 6 Unit of Occupational Medicine, IRCCS Azienda Ospedaliero-Universitaria di Bologna, Via Pelagio Palagi 9, 40138 Bologna, Italy

**Keywords:** low back pain, sexual behavior, disability, physical therapy, referral, consultation

## Abstract

Background: The literature shows a relationship between sexual activity and low back pain (LBP). The aim of this work is to provide a theoretical framework and practical proposal for the management of sexual disability in individuals with LBP. Methods: Based on a literature review, a team of specialized physical therapists developed a pattern for the management of LBP-related sexual disability. Results: A patient reporting LBP-related sexual disability may be included in one of four clinical decision-making pathways corresponding to one of the following: #1 standard physical therapy (PT); #2 psychologically informed physical therapy (PIPT); #3 PIPT with referral; or #4 immediate referral. Standard PT concerns the management of LBP-related sexual disability in the absence of psychosocial or pathological issues. It includes strategies for pain modulation, stiffness management, motor control, stabilization, functional training, pacing activities comprising education, and stay-active advice. PIPT refers to patients with yellow flags or concerns about their relationship with partners; this treatment is oriented towards a specific psychological approach. “PIPT with referral” and “Immediate referral” pathways concern patients needing to be referred to specialists in other fields due to relationship problems or conditions requiring medical management or pelvic floor or sexual rehabilitation. Conclusions: The proposed framework can help clinicians properly manage patients with LBP-related sexual disability.

## 1. Introduction

According to a biopsychosocial model, sexual health is defined as a state of physical, emotional, mental, and social well-being in relation to sexuality [1], and it should be protected and promoted also in disabled people [2]. Low back pain (LBP) describes pain between the lower edge of the ribs and the buttock. It can last for a short time (acute), a little longer (subacute), or a long time (chronic) [3]. Different authors observed some alterations of sexual life in patients complained of acute [4] and chronic [5] LBP. Decreasing frequency, arousal, and/or quality of sexual activity were reported by patients treated with conservative or surgical management [6,7]. The prevalence of sexual disability in diverse populations complaining of LBP ranges from 37% to 81% of subjects [6,8,9,10,11,12,13].

Patients suffering from chronic LBP appear more at risk of developing sexual disability, with some risk factors such as higher age, BMI, levels of depression/anxiety, lower educational level, family income, functional status, physical activity level, being unemployed, irregular menstruation, and prolonged duration of pain [12]. In addition, patients with spinal deformity are likely to have reduced sexual function related to older age, increased LBP, and increased sagittal vertical axis, with a link between sexual activity and lumbar stiffness [14]. However, no association between the severity of degenerative/disc changes and the quality of sex life in patients with LBP was observed [15].

Sexual disability in chronic conditions is related not only to pain but also to psychological dysfunctions [16], such as depression [17], activity avoidance, and rumination [10]. Moreover, a correlation between sexual functioning and relationship satisfaction (e.g., emotional intimacy, sexual intimacy, and mental health) appears. Therefore, an emerging key factor for sexual disability is the level of empathy of the couple, especially the presence of a supportive partner [18]. Patients with LBP report struggling to enable sexual activity, suffering from loss of pleasure and sexual identity, and needing support from partners and professionals to maintain their sex life [19].

In older people, the role of physical health as a predictor of sexual function is conflicting [20,21], even if the reported poor sexual function can be also attributable to the lack of a partner. Steckenrider [22] noted that as people get older, penetrative sex becomes less important. The hierarchy shifts to include more emotional intimacy like touching and fondling. This fact could help in having a different approach when comparing older people to adults with LBP-related sexual dysfunction.

All the cited studies mainly refer to heterosexual couples, but sexual disability should be considered also for nonheterosexual couples and alternative practices to vaginal sex. When examining sex life satisfaction, other sexual practices should be included, such as petting, fondling, masturbation, etc. The acronym KITOMI can be useful to properly describe all sexual activities. KITOMI is composed of the words kissing (Ki), touching (T), oral (O) stimulation of genitalia, masturbation (M), and intercourse (I) [21]. This acronym may help professionals and patients discuss sexual disability related to LBP and its management.

Sexual function is often overlooked by clinicians [10], also due to the influence of cultural and/or religious beliefs; despite this, patients would like to talk more about sexual disability, especially with their own physical therapist (PT) [19], and require indication on how to manage it properly [23,24,25]. As suggested by Korse and colleagues [26], sexual health counseling skills should be improved among professionals to enhance their abilities in managing these patients. PTs are among the professionals more involved in treating people with LBP [19], but the strategies to manage LBP-related disability are still under debate. For this reasons, the aim of this perspective manuscript is to provide a comprehensive theoretical framework and a practical proposal for the management of sexual disability in individuals with LBP.

## 2. Materials and Methods

A team comprising orthopedic manipulative physical therapists (OMPTs), experts in musculoskeletal (MC, CO) physical therapy and psychologically informed physical therapy (PIPT (SF, CV)), a PT specialized in pelvic floor rehabilitation and sexology consultant (AB), and a full professor of physical therapy (PP) was formed to review the literature and develop a proposal for the management of LBP-related sexual disability. More specifically, MC and CO performed a literature search and the selection of studies and data; SF and CV contributed to proposals relating to intervention on yellow-flag conditions; AB integrated assessment and treatment into clinical conditions requiring referral; and PP constantly supervised the proposals in light of the most recent concepts and procedures of physical therapy.

Each piece of information was accessible in an online document folder shared by all team members, organized and summarized by SF and CV, and the whole team constantly developed, discussed and implemented this project through eight online meetings, from February 2022 to November 2023. All the authors participated in writing this manuscript. Since this study had no human participants, it was not necessary to obtain approval from any ethics committee.

From February 2022 to November 2022, a narrative literature review was conducted on the main databases and team members’ personal libraries. The details of this review are presented in Appendix A. Based on the results of the literature review and the emerging lack of a comprehensive approach on this topic, the team identified and discussed the following four key concepts:

Item #1—How can sexual disability emerge in a patient undergoing physical therapy treatment for LBP?

Item #2—How can PTs classify patients with LBP regarding their sexual disability?

Item #3—What decision-making process can PTs adopt in the case of LBP and sexual disability?

Item #4—How can the practical adoption of this decision-making process by PTs by facilitated?

**Figure 1:**
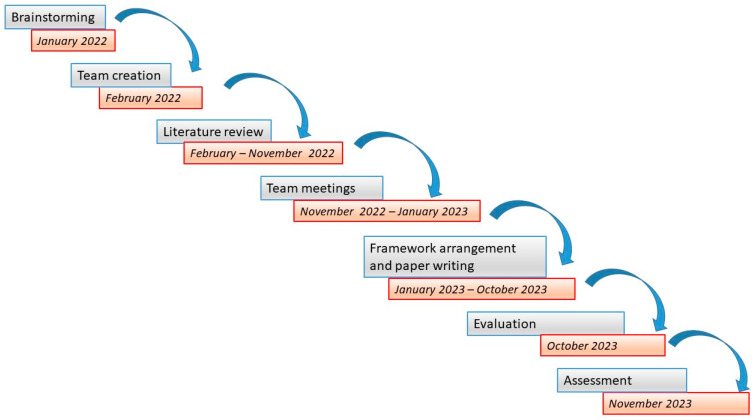
Flowchart showing the timeline of the entire project.

The results of the teamwork were discussed in a 4 h online course on “LBP and sexual disability” which took place in October 2023 and was attended by 36 PTs. The participants evaluated this proposal and judged both the framework and the indications for assessing and treating this condition to be clear and practically useful but suggested that we better explore the aspects related to PIPT. Then in November 2023, an assistant professor of physical therapy external to this research group assessed the framework by revising the manuscript, Appendix A, and Supplementary Files (see Figure 1).

## 3. Results

### 3.1. Literature Review

Patient Reported Outcome Measures (PROMs) can be administered by PTs for LBP-related sexual disability. The Oswestry Disability Index was indicated as a PROM for the disability, which includes a specific and optional item related to sexual life (ODI-8) [10,19]. The Fear-Avoidance Beliefs Questionnaire (FABQ) [27]; the Pain Catastrophizing Scale (PCS) [28] for psychosocial aspects; the Zung Depression Scale (ZDS), which contains an item related to sexual enjoyment (ZDS-6) [29]; the Beck Depression Inventory-II (BDI-II), which includes an item on the interest in sex (BDI-II-21) [30]; and the Hospital Anxiety and Depression Scale (HADS) [31] were also indicated.

Two instruments were reported to assess patients’ sexual functioning: for females, the Female Sexual Function Index (FSFI) [32], and for males, the International Index of Erectile Function (IIEF) [33].

Most therapeutic indications concern education based on biomechanical data. Concerning the relationship between sexual activity and spinal movements, two studies by Sidorkewicz and McGill [34,35] on motion capture of coitus provided indications of lumbar spine motion for males and females during sexual intercourse. Five different positions were examined: side-lying; quadruped in two variations (with female supporting on elbow; with female supporting on hands); and missionary in two variations (with male supporting on hands and female with hips and knees slightly flexed; with male supporting on elbows and female with hips and knees flexed). The results of these studies showed the amount of lumbar flexion and extension in each position and the overall amount of spinal motion during sexual intercourse.

Through these data, the same authors elaborated an ergonomic strategy, based on a classification of the five positions in three categories: flexion-intolerant, extension-intolerant, and motion-intolerant. The rationale is to inform the patient that certain positions, for males or females, may exacerbate lumbar flexion or extension and provoke or worsen symptoms, according to anamnesis and physical examination. About the “motion-intolerant” category, these authors found that all positions for males need some movement of the lumbar spine, so there is no indication in choosing one or another position; for females instead, some positions require less movement than others. Each patient should be educated to avoid positions based on which movement worsens symptoms (flexion or extension) or, for females, on how much lumbar motion they can engage in during sexual intercourse. Another strategy proposed is the use of lumbar support to decrease spinal load [34,35].

There is emerging evidence about measuring spinal movements during coitus with new tools such as an inertial device [36], which could explore better biomechanics of sexual intercourse also in LBP. We can observe that only healthy subjects were enrolled in these biomechanical studies, whereas the presence of pain during sexual intercourse could modify movement patterns and spinal range of motion (ROM). Moreover, the cited biomechanical studies are related to intercourse positions, in which it is the man who moves, whereas we did not find any study on LBP in the woman’s position on top, controlling the movement of her intercourse.

A measurement of male and female hip range of motion (ROM) during different sexual positions was also performed [37]. Intercourse positions for women require flexion (95°), abduction (32°) and mostly external rotation. For men, external rotation is dominant in all positions (40°); flexion and abduction remain in a normal ROM. Considering the regional interdependence between the lumbar spine and pelvis, a screening of hip mobility should be considered to assess the ability of the patient to reach and maintain different positions without discomfort or pain. In addition, restrictions of the hip ROM may interfere with lumbar spine motion and function in patients with LBP-related sexual disability [38].

Out of the cited ergonomic advice, the current literature lacks specific indication on rehabilitation of LBP-related sexual disability, since only suggestions based on clinical practice or common sense are proposed. For example, Nikoobath and colleagues [12] recommend avoiding painful positions or increasing physical activity in order to improve the quality of the sex life. Similar advice was reported in studies on knee, hip, or shoulder joints [39,40].

Finally, some suggestions for managing sexual disability are derived from studies on the rehabilitation of other clinical conditions. More comfortable positions [41], pillows and muscle-relaxing activities [42], pelvic floor training and sex education [43], walking [44], yoga [45], and increasing exercise capacity and self-confidence are suggested [46]. Physical exercise, both strength training and aerobic/cardiovascular training, appears an effective treatment for many aspects of sexual life: body image and self-esteem [47], sexual desire [48], sexual activity [49,50], erectile dysfunction [51], premature ejaculation [52], and depression [53]. Therefore, physical activity seems to have a positive effect on sexual function and may be considered in patients with LBP-related sexual disability.

The literature review showed the lack of a framework providing a comprehensive plan of assessment and treatment in this field for PTs. Therefore, the team elaborated and proposed an original framework for the management of LBP-related sexual disability inspired by the four pathways for decision making concerning yellow-flag management in orthopedic conditions [54]. The framework proposed by our team takes into consideration the characteristics of LBP-related sexual disability, the presence of yellow flags (e.g., fear-avoidance behavior, pain catastrophizing, kinesiophobia, or poor pain self-efficacy), the concerns about the relationship with one’s own partner, and the presence of red flags or other conditions related to specific pathologies/dysfunctions. The purpose of this algorithm is to help PTs to frame the clinical profile of each patient complaining of LBP and sexual disability and to manage sexual disability in different clinical conditions through specific assessment and treatment procedures.

### 3.2. How Can Sexual Disability Emerge in a Patient Undergoing Physical Therapy Treatment for LBP?

LBP-related sexual disability may emerge during both assessment and treatment. The PT can ask a specific question (e.g., “Does your LBP affect your sex life?”), or a patient may spontaneously report problems in his/her sex life. The administration of the Oswestry Disability Index (ODI) [55] or the Aberdeen Low Back Pain Scale (ALBPS) [56] may be another option, with particular reference to items related to sexual disability (ODI #8, ALBPS #17). After PROM completion, the PT may discuss the results of these items with both the patients who did not complete them and the patients reporting sexual disability [10].

To respect the patient’s privacy and in considering the sensitivity of the topic, the PT could introduce the discussion on sexual disability in this way: “Would you like us to talk about this?” and “I’m trying to figure out if I can help you…”.

### 3.3. How Can PTs Classify Patients with LBP Regarding Their Sexual Disability?

According to Holmberg and colleagues [11], patients with LBP can be divided into three subgroups: #1 normal sex life, without pain; #2 pain preventing any type of sexual activity; and #3 pain during sexual activity. Therefore, two symptomatic conditions may be considered: patients unable to perform sexual life because of pain, and patients reporting pain during sexual activity. The team investigated this last condition, highlighting that pain could be mainly provoked/worsened by a specific position or by changing position during sexual activity. In addition, another scenario was included, that is, when LBP emerges not during but after sexual activity. Furthermore, the team included other clinical pictures, in which pain related to sexual activity can be also referred to as yellow flags, problems in the couple’s relationship, and specific urologic, gynecologic, or andrologic pathologies.

### 3.4. What Decision-Making Process Can PTs Adopt in the Case of LBP and Sexual Disability?

The framework proposed by our team is composed of four clinical decision-making pathways corresponding to the following: #1 standard physical therapy; #2 psychologically informed physical therapy (PIPT); #3 PIPT with referral; and #4 immediate referral (see Table 1).

#### 3.4.1. “Standard Physical Therapy” Pathway

This pathway concerns the management of LBP-related sexual disability in the absence of relevant psychosocial or pathological issues. Therefore, this category includes symptomatic conditions linked to sexual activity without any presence of yellow or red flags and without any relevant influence by the relationship with one’s partner.

The Patient Reported Outcome Measures (PROMs) suggested to identify this condition and measure clinical outcomes are the ODI [55] or ALBPS [56] for disability and the Pain Self-Efficacy Questionnaire (PSEQ) [57] for predicting the patient adherence towards an active approach.

**Figure 2:**
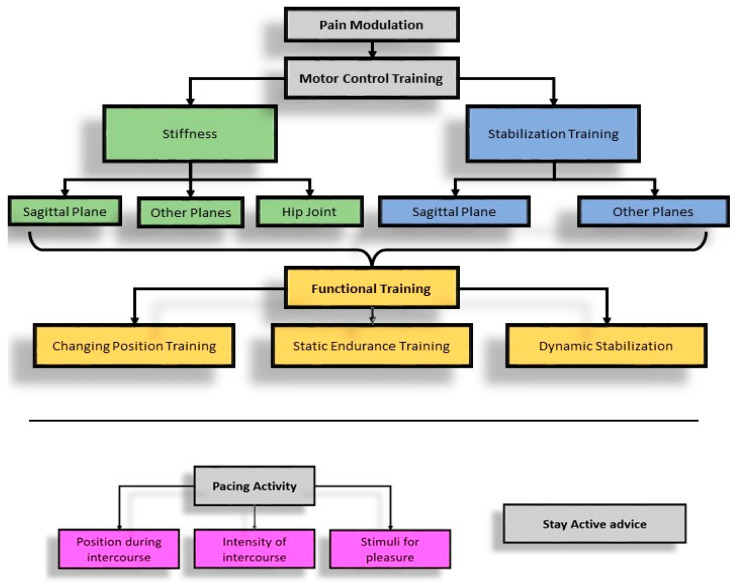
Therapeutic algorithm for standard physical therapy.

According to anamnestic collection and outcomes, different steps of rehabilitation are proposed: pain modulation, motor control training, stiffness or stabilization training, and functional training. Pacing activity and stay-active advice complete this program (see Figure 2).

Many patients report that pain prevents sexual intercourse. In this situation, pain should be reduced to enable sexual activity by using manual therapy [58,59], physical agents (e.g., transcutaneous electrical nerve stimulation) [60,61], and midrange low-load exercises. When pain is no longer inhibiting sexual activity, a PT may proceed with other steps of the therapeutic algorithm; if a patient reports no avoidance of sex because of pain, this therapeutic section can be skipped [62].

A key element of the rehabilitation program is lumbar motor control [63]. According to the studies on motion capture during coitus, through-range motor control of pelvic anti-/retroversion should be trained in the most frequent positions of sexual intercourse (e.g., supine, quadruped, prone, kneeling, seated, and standing) to allow patients to control complete lumbar ROM and increase spinal perception. The PT may use specific positions according to the patient’s evaluation [64].

Lumbar or hip stiffness could interfere with sexual intercourse and provoke/worsen pain in any position. For lumbar stiffness on the sagittal plane, flexion/extension movements are suggested; the progression may be from midrange to end-range movements and from low load (e.g., supine, prone) to half load (e.g., seated position) and full load (e.g., standing position). For lumbar stiffness in other planes, a PT may propose single-plane movements (e.g., rotation, side bending, and lateral shift), or multiplane movements. A little amount of pain should be tolerated, and the patient is encouraged to explore progressively wider ROM.

For hip stiffness, the program addresses the movements more involved in sexual activity: flexion, abduction, and external rotation [37].

Other patients need to improve spinal stabilization on the sagittal plane and/or other planes (e.g., coronal, transverse). Stabilization training is more than core muscle strengthening, involving both coordination among core muscles and motor relearning of inhibited muscles. Stabilization training in this field is an evolution of motor control training and should be performed in different intercourse positions, with the specific goal being to improve the quality of sex life [65].

The final step of this program should be functional training, which can be divided in three categories based on anamnesis: #1 “position change” (for patients reporting pain during position changing), #2 “static endurance” (for patients reporting pain while maintaining a position), and #3 “dynamic stabilization” (for patients reporting pain during/after sexual intercourse).

“Position change” training focuses on the ability to assume different positions, as usual during sexual activity, and can be trained by specific sequences of different positions with the aim of achieving pain-free movements between different sequences. “Static endurance” training is the completion of stabilization training but is more specific and progressive with regard to time and difficulty of sustained positions, with the aim of increasing the ability to stay in a position for a long time. “Dynamic stabilization” training aims to reproduce in the clinical setting the movements of sexual intercourse. Using bands, a PT can increase the force needed to complete these movements both in concentric and eccentric phases.

Pacing activity in this field is the patient’s education on how to manage symptoms during and after sexual activity. Patients can modulate sexual activity in three ways: the intercourse position, the intercourse intensity, and the stimuli for pleasure.

**Figure 3:**
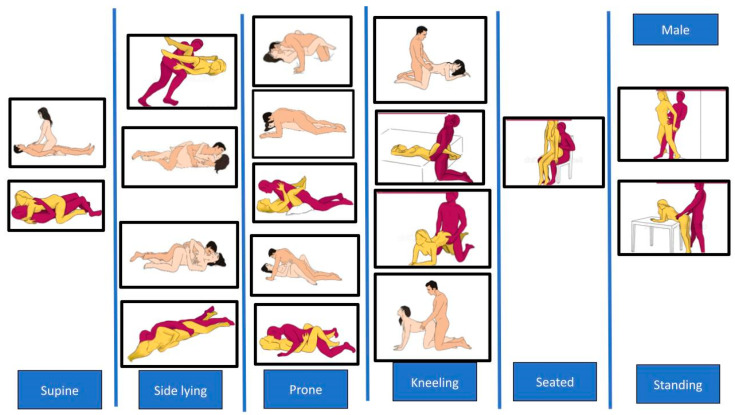
Different male positions for sexual intercourse. The pictures are taken from the websites https://www.sexualpositionsfree.com (accessed on 22 March 2023) and https://sexpositions.club/positions (accessed on 22 March 2023).

**Figure 4:**
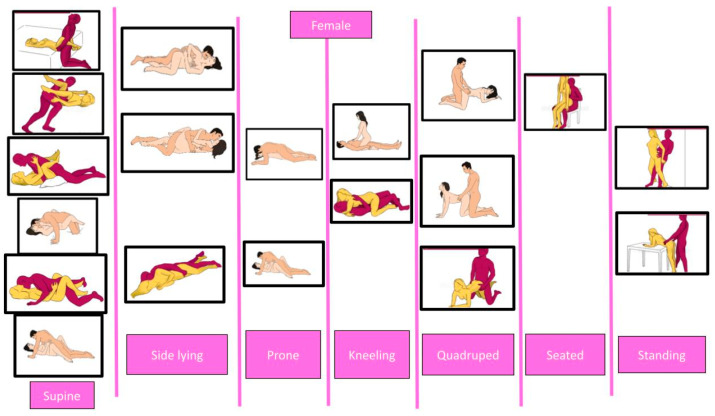
Different female positions for sexual intercourse. The pictures are taken from the websites https://www.sexualpositionsfree.com (accessed on 22 March 2023) and https://sexpositions.club/positions (accessed on 22 March 2023).

Figure 3 and Figure 4 show male and female intercourse positions from different studies [34,35,37,66], divided in supine, side-lying, prone, kneeling, seated, and standing.

**Figure 5:**
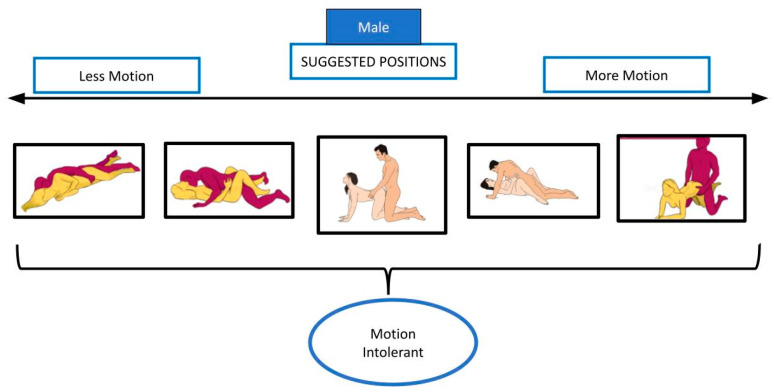
Suggested position for motion-intolerant males. The pictures are taken from the websites https://www.sexualpositionsfree.com (accessed on 22 March 2023) and https://sexpositions.club/positions (accessed on 22 March 2023).

**Figure 6:**
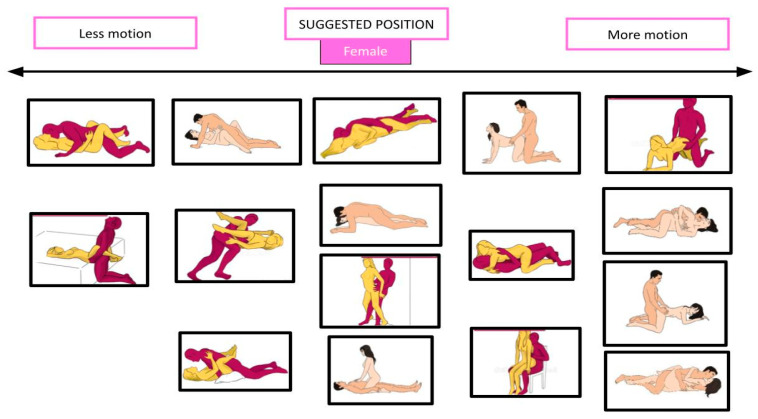
Suggested position for motion-intolerant females. The pictures are taken from the websites https://www.sexualpositionsfree.com (accessed on 22 March 2023) and https://sexpositions.club/positions (accessed on 22 March 2023).

**Figure 7:**
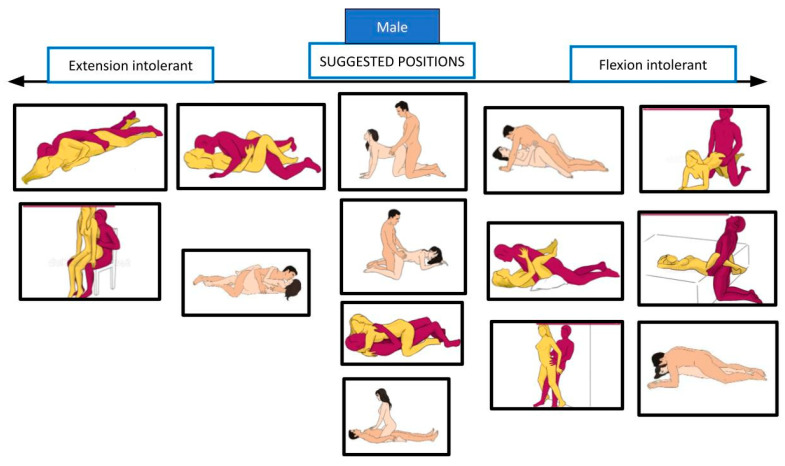
Suggested positions depending on the flexion/extension intolerance for males. The pictures are taken from the websites https://www.sexualpositionsfree.com (accessed on 22 March 2023) and https://sexpositions.club/positions (accessed on 22 March 2023).

**Figure 8:**
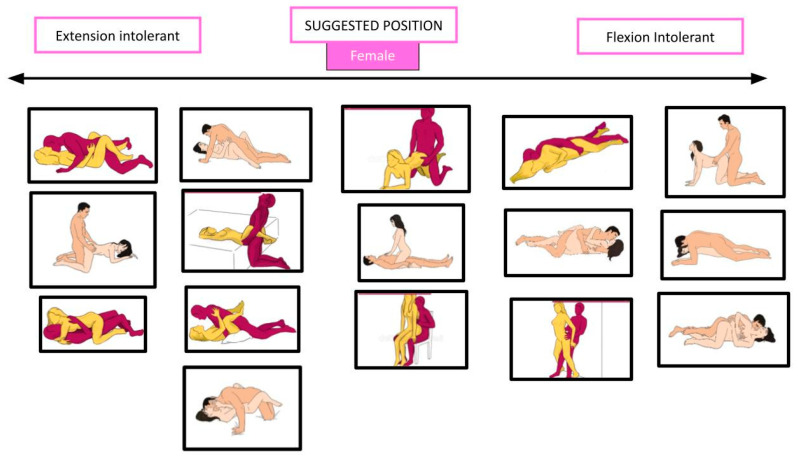
Suggested positions depending on the flexion/extension intolerance for females. The pictures are taken from the websites https://www.sexualpositionsfree.com (accessed on 22 March 2023) and https://sexpositions.club/positions (accessed on 22 March 2023).

Figure 5, Figure 6, Figure 7 and Figure 8 show the suggested positions depending on clinical picture for males and females.

First, the PT can advise using the more comfortable position and teach all variations allowed; then, positions that are more difficult may be introduced, for a short time, and alternated with positions that are more comfortable. Another strategy could be using more difficult positions at the beginning of the sexual intercourse and then use pain-free positions. The PT can advise patients on how to manage the onset or worsening of symptoms due to positioning during coitus.

Finally, stay-active advice means not only maintaining the usual activities of daily living (ADLs) and usual physical activity level but also modifying some risk factors for poor sex life related to habits. A PT can suggest patients increase their level of activity, especially aerobic (e.g., walking, cycling, Nordic walking, running, or swimming) [66,67,68,69,70]; reduce tobacco, alcohol, or drug consumption; manage sleep deprivation; and manage body mass index (BMI) and/or metabolic syndrome [71,72].

Every step of this program is related to a specific issue that may be addressed or not by a PT depending on patients’ anamnesis, physical examination, and tests/questionnaires. The focus is a tailored treatment for each single patient, applied with the graded activity and graded exposure concepts, together with stay-active advice, to restore sexual function to the maximum possible level.

More details of the therapeutic procedures proposed for this pathway are reported in Supplementary File S1 and Tables S1–S7.

#### 3.4.2. “PIPT” Pathway

This pathway refers to patients with some yellow flags (e.g., kinesiophobia, catastrophizing, fear-avoidance behaviors, and low pain self-efficacy) or concerns about the possible negative influence of sexual life on partnership in absence of evident relationship alterations.

The PROMs suggested for identifying this condition and measuring clinical outcomes are the ODI [55], the ALBPS [56], and the Optimal Screening for Prediction of Referral and Outcome Yellow Flags (OSPRO—YF) [73] as multidimensional tools and the FABQ [27], the PCS [28], the PSEQ [57], and the Tampa Scale of Kinesiophobia (TSK) [74] as unidimensional tools.

PIPT represents the attention and attitude of the PT in exploring and treating the psychosocial aspects of patients complaining of LBP, taking care of sexual disability while improving symptoms and functional limitations. The therapeutic program is similar to the “Standard Physical Therapy” one, with more emphasis on the psychosocial aspects of rehabilitation and less on the mechanical ones.

Managing this situation, it is suggested to proceed with the four steps described in the “Standard Physical Therapy” picture (pain modulation, motor control training, stiffness or stabilization training, and functional training) together with pacing activity and stay-active advice, but the treatment should be oriented towards a specific psychological approach, like pain neuroscience education (PNE) or cognitive behavioral therapy (CBT).

PNE aims to shift from the concept of pain as a portrayal of harm to the concept of pain as an alarm system for tissue protection [75]. There is some growing evidence that it could be combined with usual care in individuals with LBP to reach better outcomes [76,77]. Patients need to know about their pain that pain does not mean hurt; pain experience is multifactorial; and pain overprotective systems can be retrained [78]. For this reason, PNE should be introduced for patients presenting yellow flags.

CBT is one of the nonpharmacological therapies of choice for chronic conditions, with proven effectiveness in the management of individuals complained of LBP [79,80]. It is a psychological approach focused on removing positive reinforcement of pain behaviors and promoting problem-solving behaviors, with an additional focus on changing unhelpful cognitions [81]. This kind of therapy or similar approaches may be useful in the management of sexual disability with psychosocial components.

#### 3.4.3. “PIPT with Referral” Pathway

This pathway concerns patients mainly presenting alterations in the relationship with their partner and needing to be referred to a specialist in other fields. This condition includes patients reporting both LBP and a dissatisfaction with sexual life not necessarily related to that pain. In fact, this condition may be characterized by unsatisfactory quality/frequency of sexual activity, problems in the relationship with one’s partner, or aspects related to one’s role in the couple.

The PROMs suggested for identifying this condition and measuring clinical outcomes are the ZDS [29], the BDI-II [30], and the HADS [31] for anxiety and depression; the Changes in Sexual Functioning Questionnaire (CSFQ) for sexual dysfunctions [82]; the FSFI [32] and the IIEF [33] for females’ and males’ sexual dysfunctions, respectively [83]; and finally, the Revised Dyadic Adjustment Scale (DAS) [84] for assessing the quality of the couple’s relationship. It is suggested to refer those patients to a clinical sexologist or PT specialized in pelvic floor rehabilitation for better management of these concerns by appropriate professionals. Meanwhile, if agreed with the team and the patient, the PIPT program can proceed as presented in the previous section: like before, the focus is more on the person’s needs about sexual disability.

#### 3.4.4. “Immediate Referral” Pathway

This pathway refers to patients needing specialized assessment and treatment or reporting other sexual dysfunctions requiring pelvic floor or sexual rehabilitation.

The PROMs suggested for identifying this condition and measuring clinical outcomes are the CSFQ [82] for sexual dysfunctions and the FSFI [32] and the IIEF [33] for females’ and males’ sexual dysfunctions, respectively. If a patient reports symptoms/signs of urological, gynecological, or andrological disorders interfering with sexual activity, he/she should be immediately referred for a proper medical evaluation and treatment. In addition, the presence of sexual dysfunctions (e.g., erectile dysfunction, vaginismus, dyspareunia) need specialized assessment on their impact on sexual activity; in this case, a patient may be referred to a doctor, clinical sexologist, and/or PT specialized in pelvic floor rehabilitation. An agreement with those professionals should be sought to decide a possible return to the PIPT rehabilitation program.

### 3.5. How Can the Practical Adoption of this Decision-Making Process by PTs by Facilitated?

The team decided to elaborate and supply detailed standard physical therapy program and procedures (see Supplementary File S1), complete with images (see Supplementary Tables S1–S7), according to the Consensus on Exercise Reporting Template (CERT) checklist [85]. Four clinical cases were derived from the professional practice of the members, which could practically illustrate the process of managing different clinical conditions of sexual disability in patients treated for LBP, and are reported in Supplementary File S2.

## 4. Discussion

Rehabilitation of LBP has been widely studied; however, counseling and management of LBP-related sexual disability are underestimated. It is confirmed by the current literature, which lacks complete guidance on the assessment and treatment of this condition [86], which the patient does not perceive as an uninteresting or secondary limitation but as an actual disability [87].

It is not certain if lumbar pain reduction and lumbar function enhancement following conservative or surgical procedures will imply an improved sexual life [88,89]. Therefore, health professionals should not avoid this topic and should consider LBP-related sexual disability as a specific field of intervention. A tailored approach to sexual disability can help the patient in transferring motor abilities to a sexual relationship with his/her partner. On the other hand, a better relationship can reduce the fear of losing sexual identity and the perception of being a disabled person, finally facilitating positive thinking [87].

As recently outlined, people with motor disability require support for recovering and maintaining their sexual life [87], and healthcare professionals need appropriate training on this topic [90]. The barriers that may hinder the management of this condition can be cultural, social, religious, and educational [91], and they can concern both healthcare professionals and patients.

Based on the available evidence and current musculoskeletal approach, this paper presents a diagnostic and therapeutic framework for LBP-related sexual disability. This proposal should be adapted to each single patient and to each single PT by respecting personal beliefs and contextual variables and taking into consideration the sensitivity of this topic.

The strengths of this framework are first linked to the overcoming of a biomechanical approach emerging from the revised literature on LBP-related sexual disability towards a more comprehensive approach to care of patients, because sexual function and the consequent quality of life are closely linked to psychological, relational, and social contexts. Therefore, this framework and therapeutic algorithms move from a mechanical perspective towards a biopsychosocial model by considering all the aspects of sexual life.

This proposal can allow the identification of yellow and red flags and the drafting of a therapeutic program tailored for each patient, which can also be adapted to changes in the clinical picture over time. Moreover, physical therapy programs and education do not follow a negative approach (what a patient must avoid), as highlighted in the past literature, but a positive one (what a patient can do and how he/she can do it).

The main limitations of this study are related to the absence of a systematic literature review before the development of this framework, which may have induced potential bias in the literature search and selection. The suggested proposal may be also biased by our cultural approach to sexual health, which could limit its application in different countries and cultures. Moreover, this theoretical framework was assessed by only one expert instead of a group of experts external to the research team. Future discussion with other experts and updates/revisions are needed to validate this framework and improve its appropriateness and effectiveness.

Some areas of development of this topic may be related to the counseling about specific needs of same-sex couples and nonpenetrative sexual practices. “Sex” and “gender” concepts should be integrated in this framework [91] due to the prevalent studies on males performing vaginal sex. Another area of interest concerns the physical demands of sexual intercourse separate from the kinematic ones (range of motion and penetration circle), e.g., related to energy expenditure, heart rate, blood pressure, and perceived exertion [92]. Further studies on this topic could help to identify the volume or intensity of physical exercise focused on sexual intercourse by considering it as a form of physical activity.

Moreover, we suggest a thorough investigation of the barriers and facilitators for patients and clinicians in addressing and managing LBP-related sexual disability. Future studies could also identify more effective counseling tools (brochures, images, etc.) to be used in a physical therapy setting [90], which may be different from those commonly employed in sexual education addressed to health people.

## 5. Conclusions

Clinicians should include sexual activity within the scope of ADLs and whether a sexual disability emerges, both spontaneously and thanks to a facilitation, they should proceed on the assessment allowing the identification of the specific pathway for each patient and the administration of measures confirming or not the previous hypothesis.

Four pathways for the management of LBP-related sexual disability are proposed, together with useful algorithms for PTs in their clinical practice. It is expected that these four pathways for decision making can help PTs properly assess and treat patients with LBP-related sexual disability and finally improve the physical and psychological condition of patients through tailored strategies to manage their sexual life.

## Figures and Tables

**Table 1:** Four pathways of the decision-making process for the management of LBP-related sexual disability. Different colors refer to the usual meaning of green, yellow, orange, and red flags.

**LOW BACK PAIN-RELATED SEXUAL DISABILITY EMERGED THROUGH THE FOLLOWING:** A question asked by physical therapist: “Does your low back pain affect your sex life?” The Oswestry Disability Index (ODI) questionnaire—item #8. The Aberdeen Low Back Pain Scale (ALBPS)—item #17. Spontaneous reporting of problems in sexual life by patient.			
**STANDARD** **PHYSICAL THERAPY**	**PSYCHOLOGICALLY** **INFORMED PHYSICAL THERAPY (PIPT)**	**PIPT** **WITH REFERRAL**	**IMMEDIATE** **REFERRAL**
**Screening** LBP that prevents intercourse. LBP that does not prevent intercourse, but that appears as follows: - During intercourse (changing position; maintaining a position; during movement). - After intercourse.	**Screening** Fear of LBP (fear-avoidance behaviors). Fear of aggravating one’s condition (catastrophizing, kinesiophobia). Fear of influencing the relationship with one’s partner.	**Screening** Dissatisfaction with sexual life not related to low back pain (quality or frequency of sexual intercourse). Problems in the relationship with one’s partner. Aspects related to one’s role in the couple.	**Screening** Urological pathologies. Gynecological pathologies. Andrological pathologies. Sexual dysfunctions already diagnosed.
**Outcome Measures** - ODI (item #8) - ALBPS (item #17) - PSEQ	**Outcome Measures** - ODI (item #8) - ALBPS (item #17) - OSPRO—YF - PSEQ - TSK - PCS - FABQ	**Outcome Measures** - ZDS (item #6) - BDI-II (item #21) - HADS - CSFQ - FSFI (females) - IIEF (males) - DAS	**Outcome Measures ** - CSFQ - FSFI (females) - IIEF (males)
**Therapeutic Goals** - Pain modulation. - Stability increasing. - Endurance increasing. - Information/education. - Pain self-efficacy and self-management enhancement.	**Therapeutic Goals** - Information/education. - Pain self-efficacy and self-management enhancement.	**Therapeutic Goals** Check if the PIPT can proceed together with the care plan of the professional to whom the patient was referred.	**Therapeutic Goals** - Identified by the professional to whom the patient was referred. - Verification of a possible return to condition as pathways #3 or #2.
**Plan of Care** - Pain relief therapies (manual therapy, physical agents, exercise). - Motor control training. - Specific graded activity. - Pacing activity. - Stay-active advice.	**Plan of Care** PIPT: physical therapy procedures together with the following; - Pain neuroscience education. - Cognitive behavioral therapy.	**Plan of Care** - Referral to other healthcare professionals (clinical sexologist, pelvic floor physical therapist, etc.). - PIPT strategy (see pathway #2).	**Plan of Care** Referral to other healthcare professionals (specialized doctor, clinical sexologist, pelvic floor physical therapist, etc.).

ALBPS = Aberdeen Low Back Pain Scale; BDI-II Beck Depression Inventory-II; CSFQ = Changes in Sexual Functioning Questionnaire; DAS = Dyadic Adjustment Scale (revised); FABQ = Fear-Avoidance Beliefs Questionnaire; FSFI = Female Sexual Function Index; HADS = Hospital Anxiety and Depression Scale; IIEF = International Index of Erectile Function; LBP = low back pain; ODI = Oswestry Disability Index; OSPRO—YF = Optimal Screening for Referral and Outcome Yellow Flags; PCS = Pain Catastrophizing Scale; PIPT = psychologically informed physical therapy; PSEQ = Pain Self-Efficacy Questionnaire; TSK = Tampa Scale of Kinesiophobia: ZDS = Zung Depression Scale. Adapted from Stearns ZR et al. [54].

**Figure A1:**
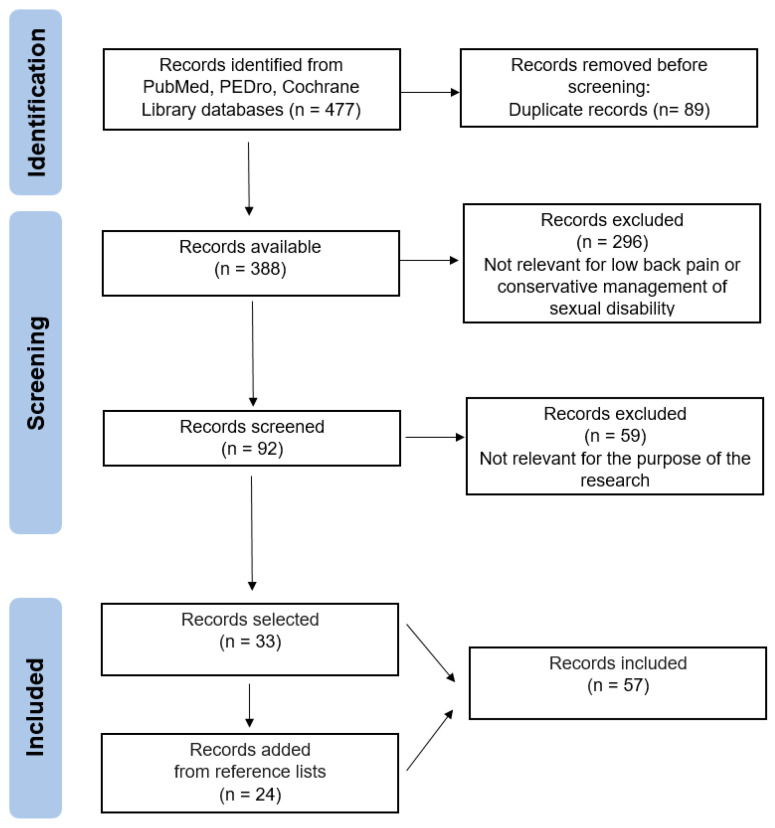
Flowchart of studies selection.

## Data Availability

No new data were created or analyzed in this study. Data sharing is not applicable to this article.

## Supplementary Materials

The following supporting information can be downloaded at: https://www.mdpi.com/article/10.3390/healthcare12010080/s1, Supplementary File S1—Standard physical therapy program: detail of the procedures; Supplementary File S2—Clinical cases.

## Appendix A. Search Strategy and Results

Key words used for search

- Sexuality, sexual activity, sexual intercourse, sexual health, coital, coitus.
- Rehabilitation, physiotherapy, physical therapy, exercise, physical exercise, physical activity.
- Low back pain, acute low back pain, chronic low back pain.

Inclusion criteria

- Types of studies: any type of study.
- Types of participants: adult population (≥18 years) with nonspecific or specific low back pain.
- Setting: any type of setting.
- Publication date: any year of publication.
- Language: any language.
- State of publication: published study.
- Types of interventions: study on assessment and/or conservative treatment of sexual disability in low back pain.
- Comparison (when applicable): different physical therapy or pharmacological interventions, wait-and-see strategies, placebo, sham, or no intervention.
- Types of outcome measures: PROMs addressing pain, disability, quality of life, sexual function, psychosocial issues.

Exclusion criteria

- Studies on population with systemic pathologies (e.g., infection, fracture, dystonia, cancer, myelopathy, rheumatic disease, fibromyalgia).
