# Supplementary material for: Sexual Disability in Low Back Pain: Diagnostic and Therapeutic Framework for Physical Therapists
Source: Healthcare (Basel). 2023 Dec 29;12(1):80. doi: 10.3390/healthcare12010080 (PMC10778778; doi:10.3390/healthcare12010080)
Supplement: Supplementary file 1 [file healthcare-12-00080-s001.zip › healthcare-2751546-supplementary.pdf]

SUPPLEMENTARY FILE 1

STANDARD PHYSICAL THERAPY PROGRAM—DETAILS OF THE PROCEDURES

S1. Pain modulation

This program is addressed to patients referred because their pain prevents sexual intercourse.

S1.1. Manual therapy can be delivered in different ways: lumbar angular passive movements, lumbar/pelvic/pubic accessory passive movements, HVLA, traction, and myofascial treatment.

S.1.2. Physical agents like TENS may be used to reduce symptoms and disability in some conditions.

S.1.3. Exercise should be more oriented to reduce pain and less to increase lumbar spine range of motion (ROM). For this purpose, midrange low-load exercises are recommended, preferably pain-free (or with a minimum presence of pain, based on patient tolerance). A high number of repetitions is suggested, with progressive ROM and load increasing. Specific attention should be given to train positions used during sexual intercourse (see Table S1).

Table S1. Exercises for pain modulation.

Description: Basic exercise with general active mobilization of lumbar spine in different directions. The aim is to reduce pain in the spine and restore mobility of lumbo-pelvic district. High number of repetitions with low loads (e.g., 3x25, 4x20) or isometric hold of the position (up to 40 seconds) are suggested.	
Supine Bridge	***Bird/Dog***
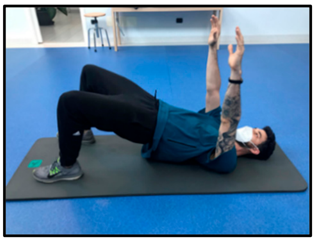	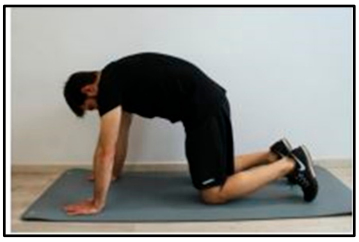
Lying supine, lift the pelvis until hip and lumbar extension, if tolerated.	In quadruped position, alternate spinal flexion/extension with focus on pelvic anti-/retroversion.
Unilateral Hip Flexion	***Lumbar Rotation***
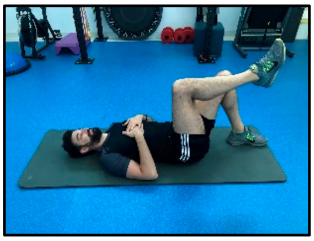	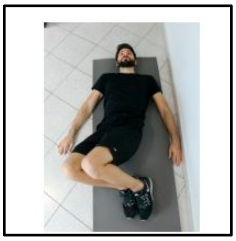
Lying supine with knee bent and feet on the floor, lift one leg by hip flexion.	Lying supine with knees bent and feet on the floor, rotate from one side to the opposite one, keeping the upper quadrant stable on the floor.

Bilateral Hip Abduction	*Lumbar Extension*
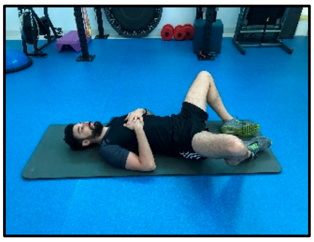	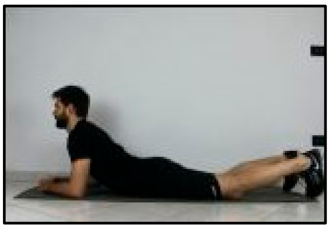
Lying supine with knee bent and feet on the floor, open legs by pushing the knees towards the floor.	Lying prone, lift the upper quadrant to tolerated lumbar extension.
Piriformis Stretch Position	***Lumbar Side Bending***
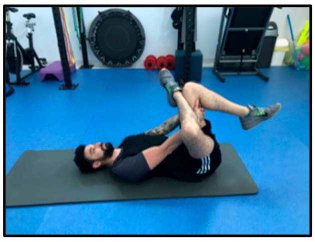	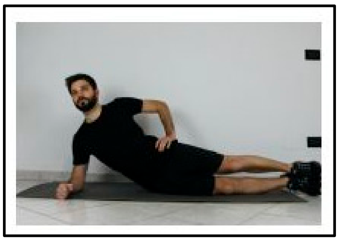
Lying supine, cross one leg to the opposite side; grab the opposite side with fingers and then slowly flex the hip until pain limit. Hold this position for at least 30–40 seconds.	Lying on one side, lift the upper quadrant with support of elbow and forearm. The opposite hand stabilizes the pelvis.

S.2. Motor control training

Motor control trains anti-/retroversion movements of lumbo-pelvic district in different positions of sexual intercourse. This training focuses more on control of through-range lumbar movements than of the neutral zone. The aim is to develop the ability to modulate lumbar flexion/extension during sexual intercourse: patient should be able to manage onset/worsening of pain by adjusting spinal position and movement (See Table S2).

Table S2. Training for motor control.

Description: This training aims to improve the ability to move from "neutral zone" to anti-/retroversion of the lumbo-pelvic region. The positions given are the most studied for sexual activity. A high number of repetitions is suggested.	
Supine	***Quadruped***
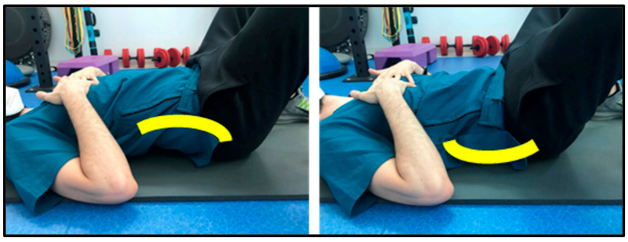	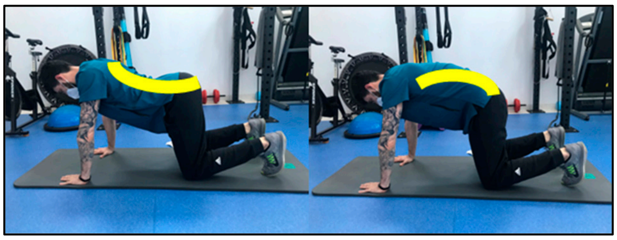
Prone	***Kneeling***

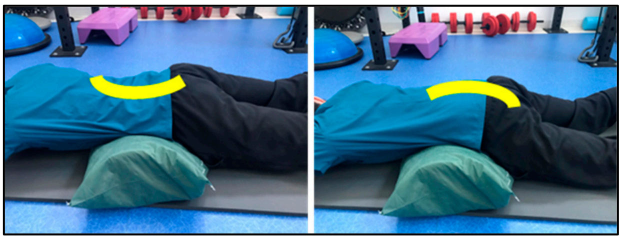	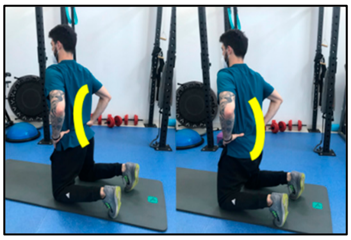
Seated	*Standing*
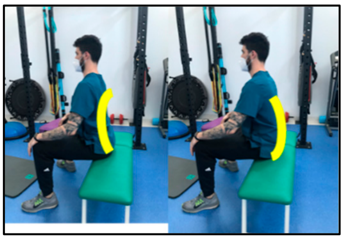	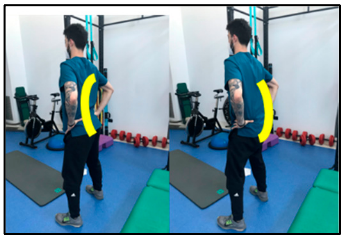

S.3. Stiffness management

Only patients with spinal or hip stiffness will be interested in this part of program.

S.3.1. Manual therapy

The same manual therapy approach suggested for pain modulation can be used to increase mobility when applied in specific limited directions.

S.3.2. Exercise

Active exercise starts from correct positioning and progresses to the end-range position; a high number of repetitions with progressive tension at the end of ROM or holding position for progressive time is suggested. Load and ROM progression are suggested: from midrange to end-range movements and from low-load (e.g., supine, prone) to half-load (e.g., seated) and full-load (e.g., standing) positions. A little amount of pain should be tolerated and the patient encouraged to progressively exploring wider ROM.

Spine stiffness can be treated on sagittal plane (see Table S3) or other planes (e.g., transverse, coronal).

Table S3. Exercises for stiffness on sagittal plane.

Description: This training aims to restore complete range of motion of lumbar flexion and extension. This training progresses from midrange to end-range exercises and from low load to seated (half load) and standing (full load). A high number of repetitions is suggested.	
Extension	***Flexion***
Midrange—Low Load	
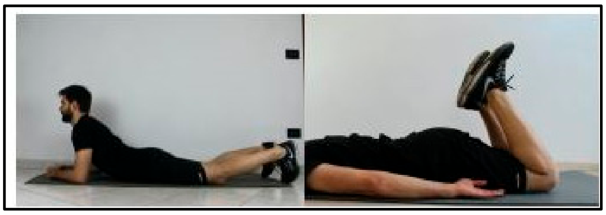	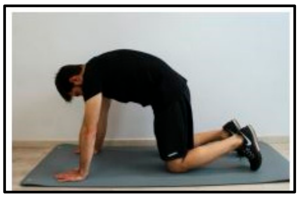

End Range—Low Load	
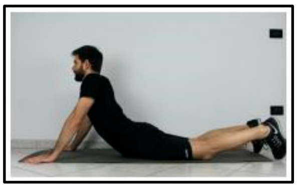	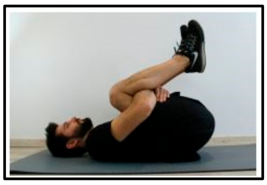
Midrange—Seated (Half Load)	
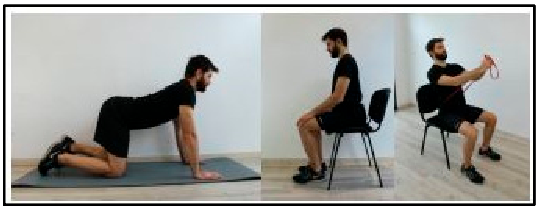	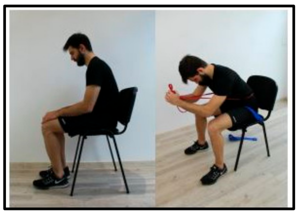
End Range—Seated (Half Load)	
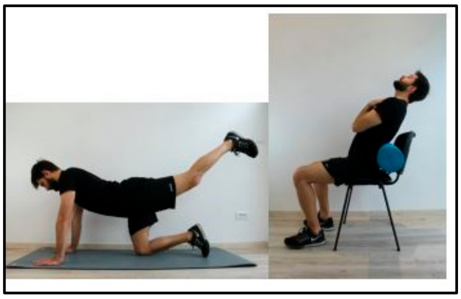	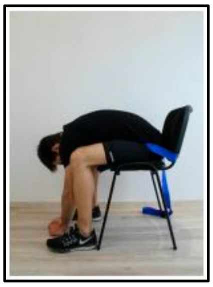
Midrange—Standing (Full Load)	
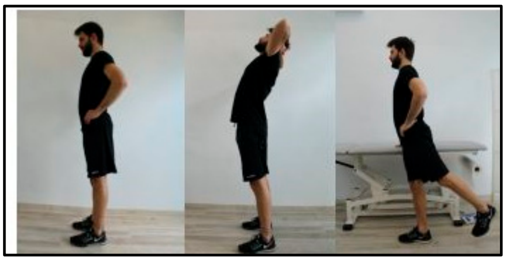	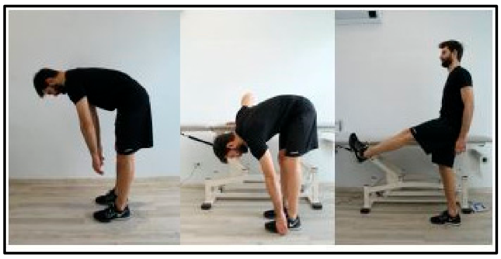
End Range—Standing (Full Load)	
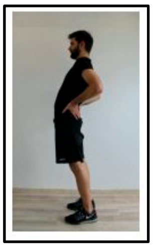	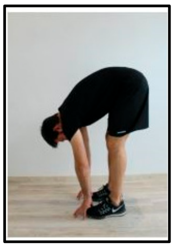

For stiffness in other planes, the program can be divided in two sections: single-plane movements and multiplane or three-dimensional movements. Single-plane items include mobility training in rotation, side bending, or lateral shift, with load progression and from midrange to end range. Three-dimensional movements are a combination of extension/flexion with rotation and side bending performed in low-load or full-load position, as in the following examples:

a) Flexion–side bending–rotation;
b) Extension–side bending–rotation;
c) Flexion–side bending–opposite rotation;
d) Extension–side bending–opposite rotation (see Table S4).

Table S4. Exercises for stiffness on planes different from sagittal.

Description: This training aims to restore complete range of motion in rotation, side bending, and side shift. Movements can be combined for three-dimensional movements. A progression from midrange to end-range exercise and from low-load to full-load positions (e.g., standing) is suggested. A high number of repetitions is also suggested.	
Lateral/Rotational Movements	
End-Range Rotation (Low Load)	***Midrange Rotation (Half Load)***
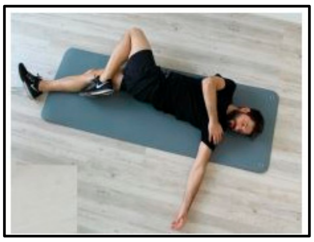	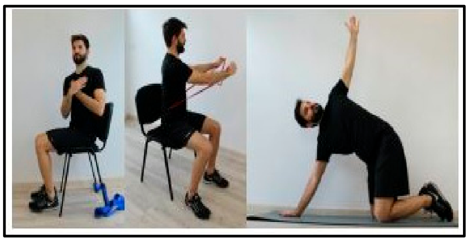
Side Shift (Full Load)	***Side Bending (Full Load)***
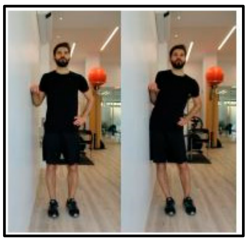	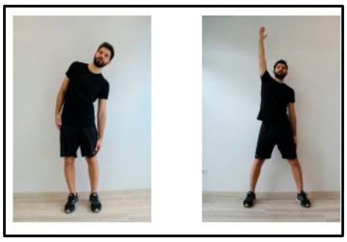
Three-Dimensional Movements	
**Low Load**	***Full Load***
Flexion, Side Bending, and Ipsilateral Rotation	
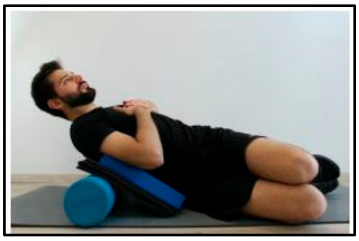	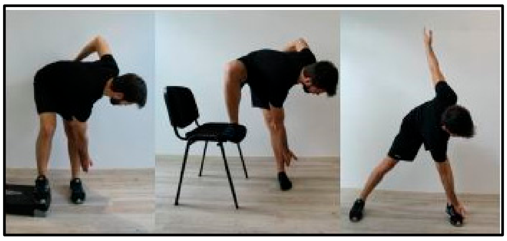
Extension, Side Bending, and Ipsilateral Rotation	
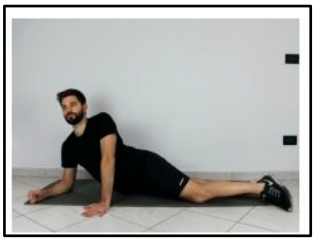	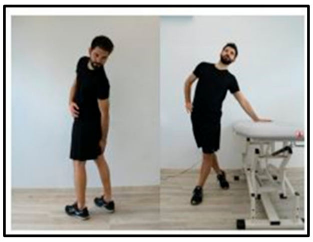

Flexion, Side Bending, and Contralateral Rotation	
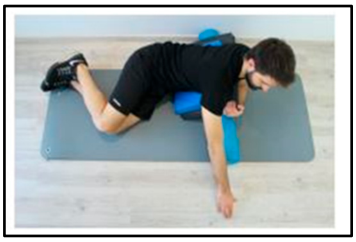	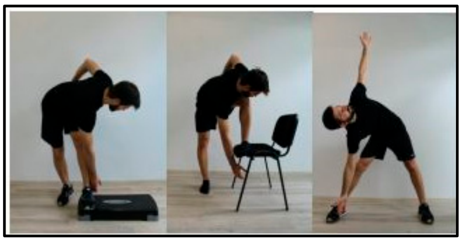
Extension, Side Bending, and Contralateral Rotation	
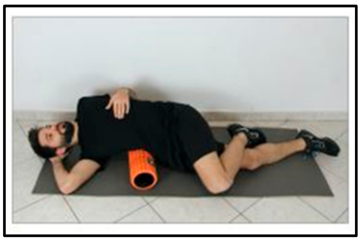	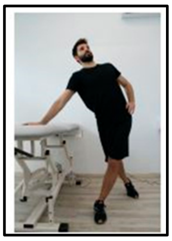

For hip stiffness, the program addresses the three movements more involved in sexual intercourse: flexion, abduction, and external rotation.

Flexion

The goal is to achieve a flexion at least of 95°, using the following:

a) Thomas test position for concentric hip flexion or passive hip flexion;
b) Sustained hip flexion in a kneeling position;
c) Passive hip flexion from quadruped position;
d) Active hip flexion from supine.

Abduction

The goal is to achieve an abduction at least of 32°, using the following:

a) Hip active abduction from supine;

b) Lateral lunge in standing;

c) Hip abduction in side lying, with leg flexed or straight;

d) Frog position with hip abducted as far as possible.

External Rotation

The goal is to achieve an external rotation at least of 40°, using the following:

a) Active internal/external rotation seated on the floor;

b) A 90/90 position, with one leg in flexion and external rotation and the other in abduction and internal rotation;

c) Active internal/external rotation from supine, with 90° hip flexion;

d) Active external rotation in side-lying position.

Therapeutic principles should be the same as for lumbar stiffness. See details in Table S5.

Table S5. Exercises for hip joint stiffness.

<table>
<tr><td colspan="2">Description: This training addresses the restoration/maintenance of hip joint range of motion, especially flexion (up to 95°) and abduction (up to 32°) for females, and external rotation (up to 40°) for males. The use of both isometric and concentric/eccentric contraction is suggested.</td></tr>
<tr><td colspan="2">Hip Flexion</td></tr>
<tr><td>Thomas Test Position</td><td>Kneeling</td></tr>
<tr><td>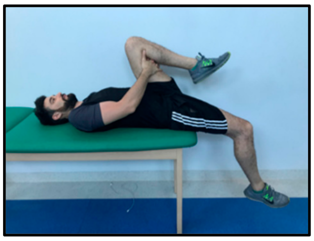
Active flexion followed by passive flexion of hip.</td><td>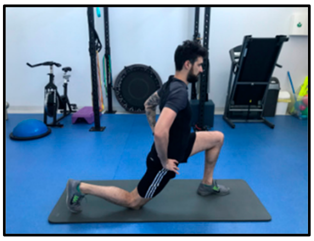
Hold position (30-40 seconds).</td></tr>
<tr><td>Quadruped</td><td>Supine</td></tr>
<tr><td>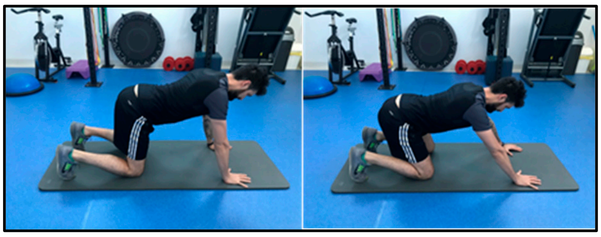
Posterior slide to increase hip flexion; hold position (30-40 seconds).</td><td>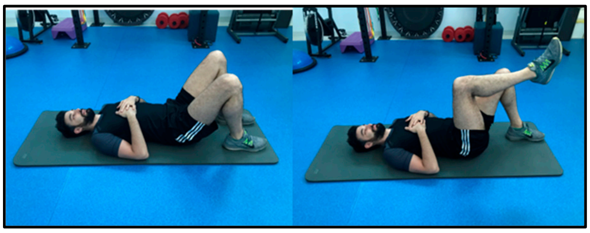
Concentric or isometric hip flexion.</td></tr>
<tr><td colspan="2">Hip Abduction</td></tr>
<tr><td>Supine</td><td>Lateral Lunge</td></tr>
<tr><td>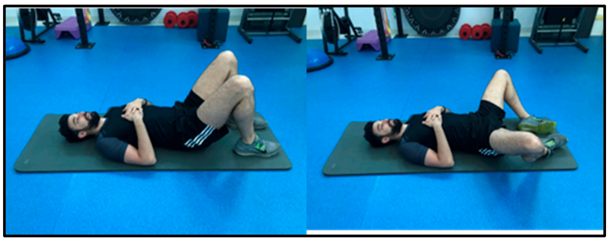
Concentric or isometric hip abduction.</td><td>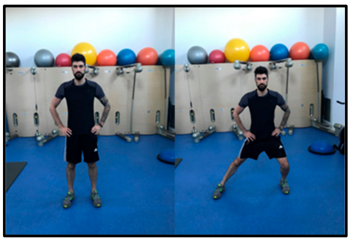
Focus on the width of lunge; it can be used in a static way.</td></tr>
<tr><td>Side Lying</td><td>Quadruped Frog</td></tr>
<tr><td>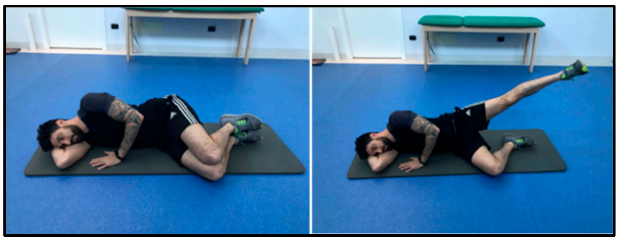</td><td>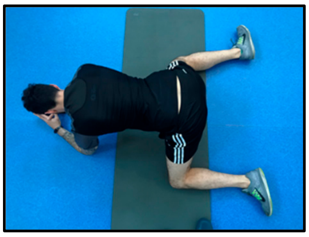</td></tr>
</table>

Concentric or isometric hip abduction.	Hold position (30-40 seconds).
Hip External Rotation	
Seated, Hip Flexed	***90/90***
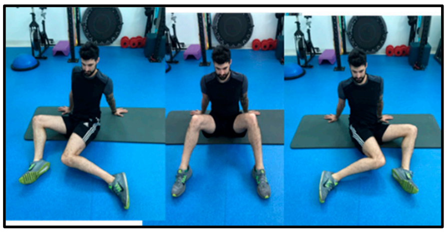 Alternatively move from internal to external rotation.	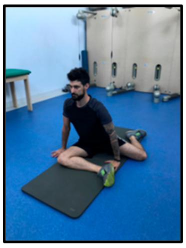 One leg is positioned in flexion/external rotation; other leg is in abduction/internal rotation. Hold position for 30–40 seconds. To increase external rotation range of motion, patient can push the knee down to the floor actively or passively.
Supine, 90° Flexion	***Side Lying***
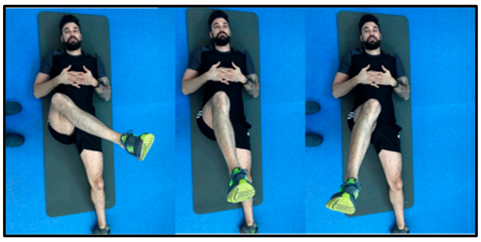 Movement from internal to external rotation at 90° hip flexion.	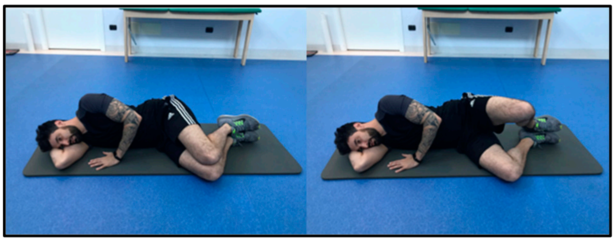 Hip flexed, lift the knee while maintaining the spine in neutral position.

S4. Stabilization training

This concerns patients needing to improve spinal stabilization, which can be divided, as for stiffness, into sagittal plane and other planes (e.g., coronal, transverse).

Stabilization training in this field should especially consider motor control in sexual positions. Physical therapists (PTs) can explore different positions, destabilization strategies, and difficulty levels by considering the specific requirements of each patient. This program involves different positions (e.g., supine, quadruped, seated, kneeling, and standing).

For stabilization on the sagittal plane, an exercise with a roller, moving upper or lower limbs to increase lumbar recruitment, is suggested. Further details are in Table S6.

Table S6. Stabilization Exercises.

Description: This training aims is to enhance lumbar stability in different positions, tasks, and destabilization stimuli. It is suggested that this program be chosen based on the patient's reported difficulties in sexual intercourse, choosing appropriate position, load, and set/repetitions.	
Sagittal Plane	
Hip Hinge	***Supine Bridge***

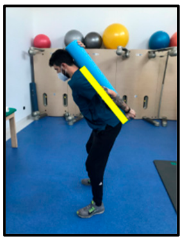	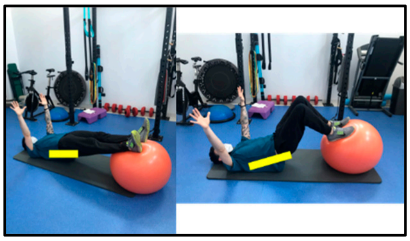
Maintain spine in neutral position while bending the trunk forward.	Use an unstable surface (e.g., fitball) and more challenging positions to increase muscle recruitment.
Standing	***Supine***
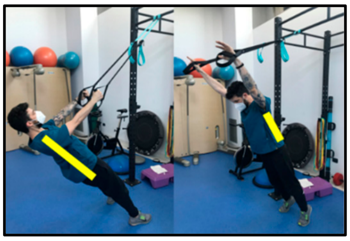	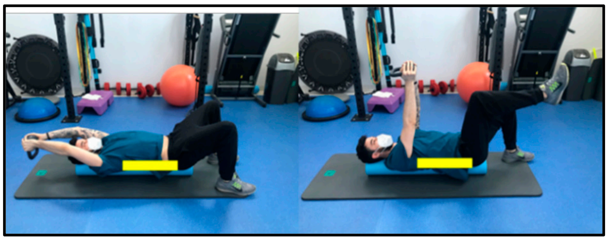
Hold static positioning while maintaining spine in neutral position.	The use of a roller increases stability demand, and upper/lower limb movements further increase lumbar muscle recruitment.
Other Planes	
Supine	***Quadruped***
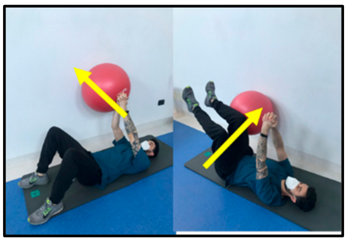	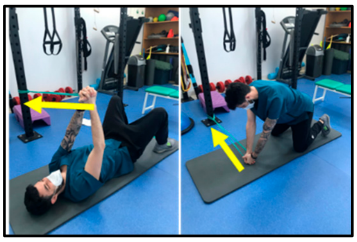
Destabilization may come from upper or lower limbs.	Destabilization comes from upper limbs, using a band. Focus on isometric, concentric, or eccentric training.
Kneeling/Seated	***Standing***
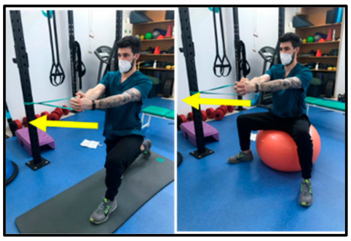	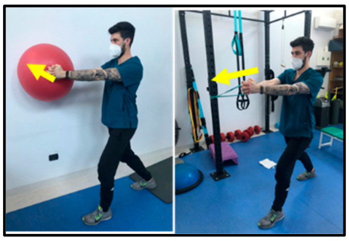
Using a band, patient can hold position or focus the training on concentric/eccentric phase.	In standing or lunge position, patient can use fitball (the patient must push) or band (the patient must resist).

S.5. Functional training

Functional training is divided in three categories based on anamnesis: #1 “position change” (for patients reporting pain during position changing); #2 “static endurance” (for patients reporting pain while maintaining a position); and #3 “dynamic stabilization” (for patients reporting pain during/after sexual intercourse) training.

S.5.1. Position Change

This functional training is addressed to patients struggling with position change during sexual intercourse, with the aim to develop the maximum ability to change position without pain or fatigue. This skill can be trained by specific sequences of selected different positions, focusing on movement control (e.g., ROM, speed, and lumbar and limb positioning) during the changing phase.

Different sequences with different difficulty levels are proposed:

a) Supine, side lying, prone, sphynx, quadruped, full flexed in quadruped, quadruped, kneeling, standing;

b) Prone, quadruped, kneeling, standing;

c) With fit ball: prone with leg flexed, prone with leg extended;

d) With fit ball: seated, supine bridge, full hip flexion, full back extension.

Every sequence can be performed partially, in reverse, or going and returning; a patient is asked to stay in a position for different times before changing it. The applied load should be incremental, including the use of any unstable surface, weights or bands, or increased time of execution.

PTs can introduce specific positions based on anamnesis or patient requirements or modify the difficulty level based on patient ability. If some positions are unreachable, patients should be taught about their use during sexual activity.

Detailed proposal can be found in Table S7.

Table S7. Functional Training: Position Change.

<table>
<tr><td colspan="3">Description: The functional training program is divided into three different tasks: position change, static endurance, and dynamic stabilization. “Position change” exercises are sequences training the ability to change position, challenging the through-range motor control of the lumbar spine.</td></tr>
<tr><td colspan="3">Position Change</td></tr>
<tr><td colspan="3">Every sequence can be performed a) partially; b) in reverse; and c) adding any other specific position or movement.</td></tr>
<tr><td>From Prone to Standing
This sequence addresses most of the positions studied in sexual intercourse.</td><td>1) Prone.
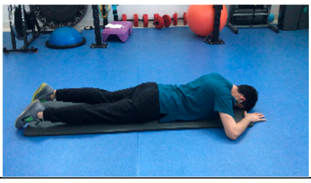</td><td>2) Quadruped.
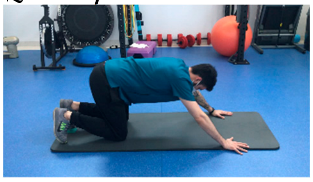</td></tr>
<tr><td>3) Kneeling.
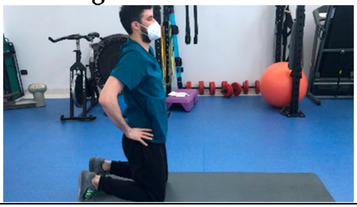</td><td>4) Lunge.
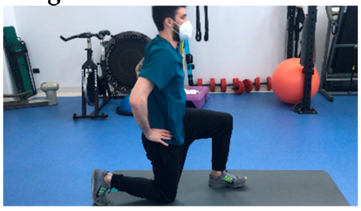</td><td>5) Standing.
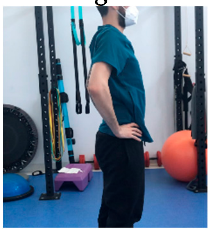</td></tr>
</table>

From Supine to Standing This sequence adds two more variations of the previous exercise.	***Supine.*** 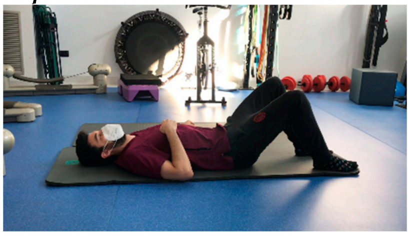	***Side lying.*** 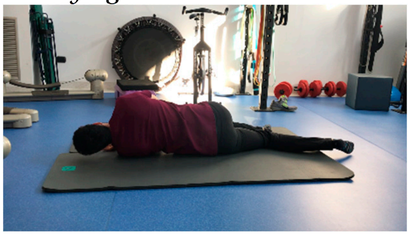
Prone. 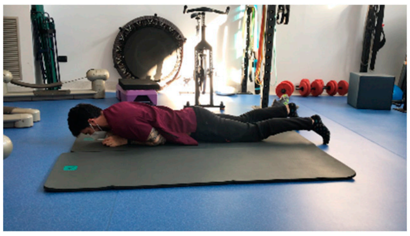	***Sphynx.*** 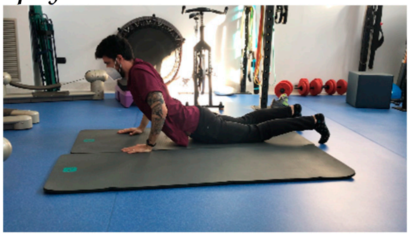	***Fully flexed in quadruped.*** 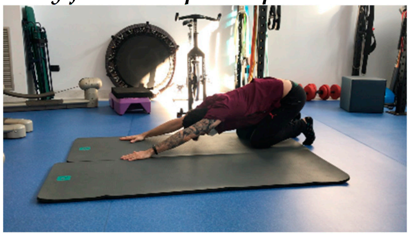
Quadruped. 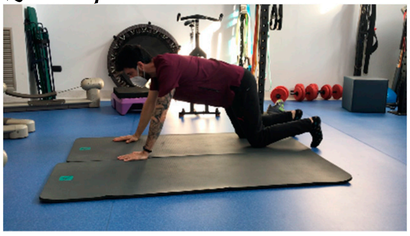	***Lunge.*** 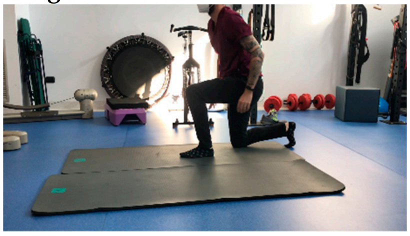	***Standing.*** 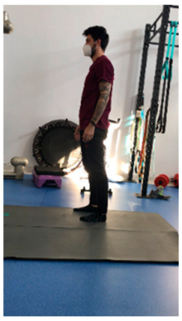
Seated on Fitball Using an unstable surface to emphasize motor control ability, focusing on pelvic stability and hip motion from flexion to extension.	*A) Seated.* 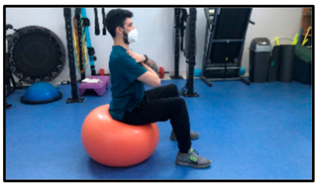	*B) Supine.* 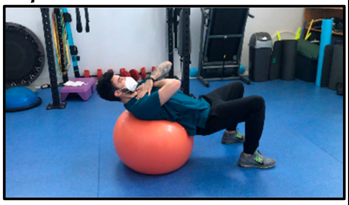
	C) Full hip flexion. 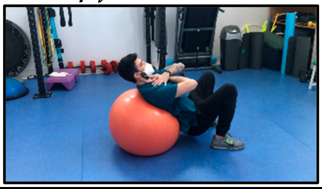	*D) Full back extension.* 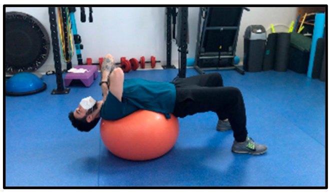
Leg on Fitball Using an unstable surface, focus on motor control of lumbar spine from flexion to extension.	*A) Leg flexed.* 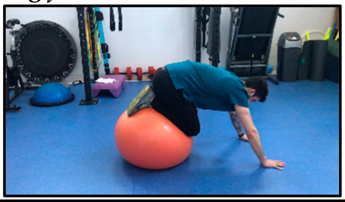	*B) Knee extended.* 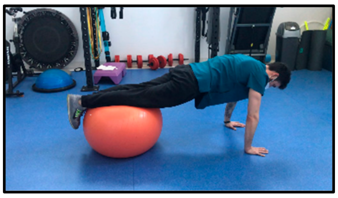

S.5.2. Static Endurance

Static endurance training should be specific and progressive for time and position. The goal is to train the patient in staying in a position for the maximum time allowed (one repetition for maximum time). Training should be performed in three positions, with three variations: supine bridge, lateral bridge, and prone bridge.

Variations of supine bridge are as follows:

a) Bilateral, knee flexed;

b) Bilateral, knee flexed on a bench;

c) Bilateral knee extended on a bench.

A weight can be used to increase fatigue. Suggested time progression is 10 repetitions x 10 seconds; 8 x 15 seconds; 6 x 20 seconds; 5 x 30 seconds; 4 x 45 seconds; 3 x 1 minute; 1 repetition x maximum time.

Variations of lateral bridge are as follows:

a) Leg flexed, elbow on the floor;

b) Leg extended, elbow on the floor;

c) One leg extended, one leg lifted in flexion, elbow on the floor.

Prone bridge variations are as follows:

a) Plank with 45° angulation;

b) Plank on elbows;

c) Plank on hands.

Suggested time progression for both lateral and prone bridge is 10 repetitions x 6 seconds; 8 x 10 seconds; 6 x 15 seconds; 5 x 20 seconds; 4 x 30 seconds; 3 x 45 seconds; 1 repetition x maximum time.

Detailed proposal can be found in Table S8.

Table S8. Functional Training—Static Endurance.

<table>
<tr><td colspan="3">Description: The functional training program is divided into three different tasks: position change, static endurance, and dynamic stabilization. “Static endurance” exercises are an evolution of the stabilization program, with three variations: supine, lateral, and prone bridge.</td></tr>
<tr><td colspan="3">Static Endurance</td></tr>
<tr><td colspan="3">Supine Bridge</td></tr>
<tr><td colspan="3">Hold position, possibly with overload.
Progression: 10 reps x 10 seconds; 8 x 15 sec; 6 x 20 sec; 5 x 30sec; 4 x 45; 3 x 1 minute; 1 x maximum.</td></tr>
<tr><td>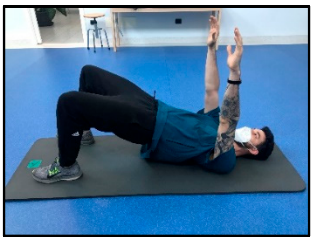</td><td>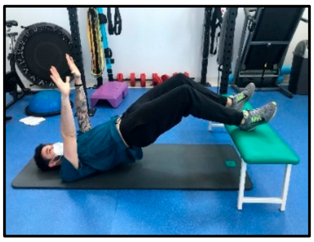</td><td>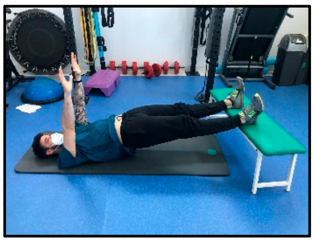</td></tr>
<tr><td colspan="3">Lateral Bridge</td></tr>
<tr><td colspan="3">Hold position, possibly with overload.</td></tr>
</table>

Progression: 10 reps x 6 seconds; 8 x 10 sec; 6 x 15 sec; 5 x 20 sec; 4 x 30; 3 x 45; 1 x maximum.		
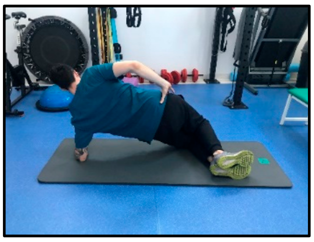	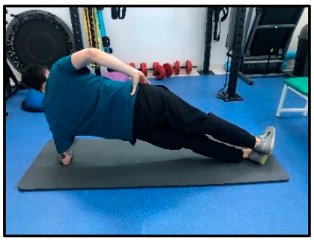	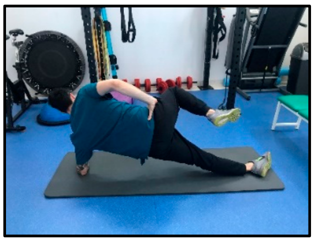
Prone Bridge		
Hold position, possibly with overload.<br>Progression: 10 reps x 6 seconds; 8 x 10 sec; 6 x 15 sec; 5 x 20 sec; 4 x 30; 3 x 45; 1 x maximum.		
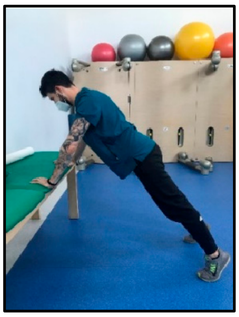	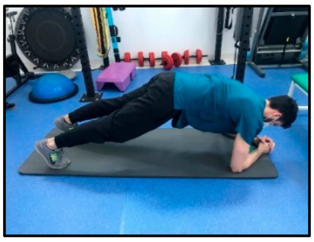	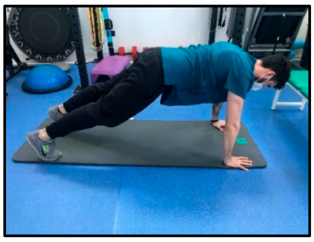

S.5.3. Dynamic Stabilization

This reproduces in a clinical setting the movements of sexual intercourse. Bands are used to increase the force in both concentric and eccentric phases. A band fixed behind the patient induces a back force opposite to muscles involved in going forward (penetration), whereas a band fixed forward to the patient induces an anterior force, synergic to muscles involved in copulation motion. A PT can focus on three different tasks:

a) Isometric: Hold position for progressive time, also increasing band resistance. It could be interesting to allow a little oscillation in small ROM to train stabilization through-range;

b) Strength: concentric phase, increasing band resistance;

c) Power: Concentric phase, increasing speed of movement and band resistance. Different training positions are suggested: supine bridge, kneeling, lateral bridge, prone bridge at 45°, hip hinge, or any other specific position or task may be tailored to patient needs.

Detailed proposal can be found in Table S9.

Table S9. Functional Training—Dynamic Stabilization.

Description: The functional training program is divided into three different tasks: position change, static endurance, and dynamic stabilization. "Dynamic stabilization" exercises train the ability of modulate lumbar movements in a similar way to the sexual intercourse.
Dynamic Stabilization
Elastic Band, Resistance Directed Posteriorly

The posterior force increases recruitment of "pushing" muscles, and variations may involve range of motion (more or less forward and backward), speed of movement (more or less forward and backward), and frequency (repetitions/time).	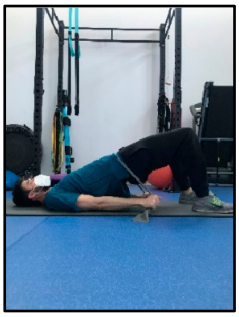	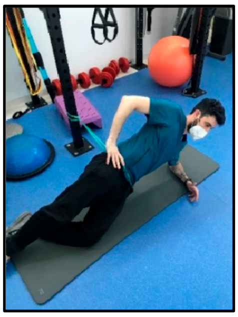
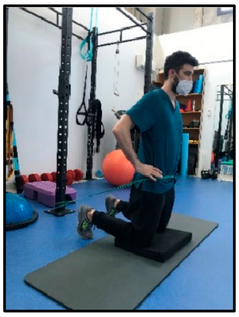	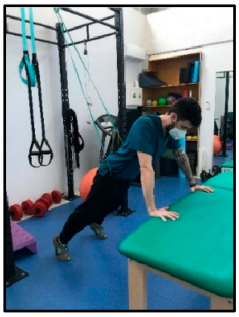	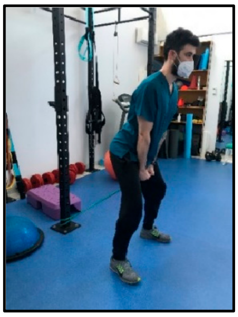
Elastic Band, Resistance Directed Anteriorly		
The anterior force reduces the work of "pushing" muscles and focuses on the eccentric phase of the antagonists. Variations may involve range of motion (more or less forward and backward), speed of movement (more or less forward and backward), and frequency (repetitions/time).	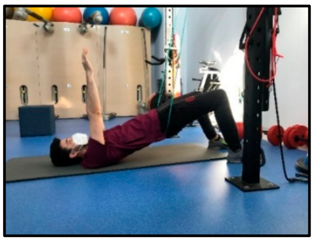	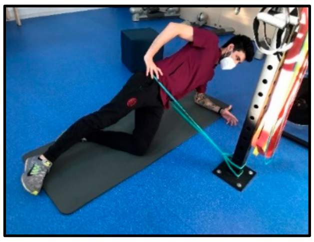
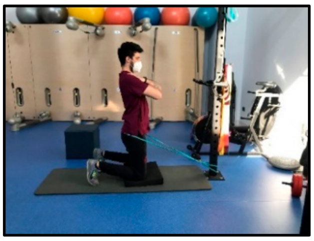	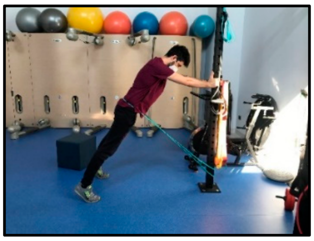	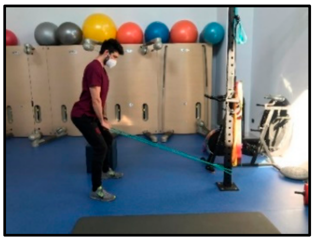

S.6. Pacing Activity

The aim is teach the patient how to manage symptoms during and after sexual activity, in three possible ways:

#1. Advice to use position that is more comfortable and teach all variations allowed (e.g., a patient with low load capacity cannot tolerate seated or standing position and can benefit from supine, side-lying, or prone ones);

#2. Introduction of more difficult positions, for a short time, alternated with positions that are more comfortable;

#3. Using the more difficult positions at the beginning of sexual intercourse and then using pain-free positions.

PTs should advise which position to use instead of which position to avoid to develop self-efficacy and motivation in patients. There is no indication about specific positions for the motion-intolerant pattern in males, since every position requires lumbar spine movement. In this case, a PT may advise males to use the more comfortable position and reduce their movement, helped by the patient's partner; then, more challenging positions could be achieved, increasing load

capacity (see Figure 4). Also for females, some possibly variations during sexual intercourse may be proposed in case of a motion-intolerant pattern (see Figure 5).

About the flexion/extension-intolerant pattern, a PT should suggest which positions to use instead of which positions to avoid; variations can help to progressively modify load and difficult of sexual activity to gradually increase patients' abilities (see figures 6,7).

Management of intensity of sexual intercourse is not simple; we propose to use two models to teach patients how to modulate intensity.

"Rule of ten" Model

According to this rule, the sum of pain (measured with the Numerical Rating Scale) and perceived effort of sexual activity (measured with the Rate Perceived Effort) must be 10 points. It is derived from the pain-monitoring model, implemented with the Borg Scale to link pain intensity and perceived effort. Therefore, patients reporting high levels of pain are allowed to perform low-intensity sexual activity, whereas patients with no/little pain are allowed to maximize the intensity of sexual activity. This model may be useful for patients who were unable to perform sexual activity due to pain because it is a simple way to self-monitor the pain trend during intercourse. It could also be a tool to teach how to introduce new positions (that were limited or too painful), because it stimulates the ability to self-evaluate the pain trend and allows patients to independently manage their sex life.

"Three-traffic-lights rule" Model

This corresponds to three steps of evaluation of the intensity and load tolerance, in this case of sexual intercourse. It is derived from the three steps of pain-monitoring model, using a traffic-lights system for decision-making support.

- The first step is answering the question "pain during activity was tolerable?" If patients say yes, this corresponds to a "green light", and he/she can go on.
- The second step is answering the question "pain after activity was tolerable?" This question addresses the pain trend over time, which could be a topic to discuss with the patient.
- If the answer is yes, proceed with the last step, asking, "did you feel more pain than usual the day after sexual intercourse?"

If the three answers are yes, the intensity of intercourse is optimal, and load can be increased.

For pleasure of intercourse, different techniques could be added during vaginal and anal penetration. You may address not only the importance of the position and the intensity of sexual intercourse (skills that you can train in clinical settings) but also information on making sexual intercourse more pleasurable.

#1. Angling

Rotating, raising, or lowering the pelvis/hips during penetration to adjust where inside the vagina the toy or penis rubs and what it feels like. This can be a strategy to use a pain-free position and not lose pleasure or a strategy to reduce pain in a painful position; the ability to modify pelvic orientation is a skill trained in the motor control phase of the physical therapy program.

#2. Rocking

The base of a penis or sex toy rubbing against the clitoris constantly during penetration, by staying all the way inside the vagina rather than thrusting in and out.

#3. Shallowing

Penetrative touch just inside the entrance of the vagina—not on the outside, but also not deep inside—with a fingertip, sex toy, penis tip, tongue, or lips. Rocking and shallowing can be useful in reducing penetration intensity and so the width and fatigue of movement during intercourse; it can be very important for those patients struggling during intercourse with low load tolerance and weak conditioning.

#4. Pairing

This occurs when a woman herself (solo pairing) or her partner (partner pairing) reaches down to stimulate her clitoris with a finger or sex toy at the same time as her vagina is being penetrated. Pairing could be a strategy to make sexual activity more pleasurable independent of position and intensity.

SUPPLEMENTARY FILE 2

CLINICAL CASES

Four clinical cases illustrate the process of managing different clinical conditions of sexual disability in patients treated for LBP.

- **Clinical case #1 – "Standard Physical Therapy" pathway**

History

A 38 year-old man reports a 2-month history of LBP, located in the back central area without any referred pain in the legs. This pain is progressively worsening over time but remains intermittent and mechanical in nature. His symptoms worsen in the seated position, after two hours standing, after dynamic activities like swimming or running, and during sexual intercourse.

Outcome Measures

NRS = 5; ODI = 22 % (Item 8: My sex life is normal but causes some extra pain); PSEQ = 30/60.

Physical Exam

The postural assessment shows a flat lumbar spine. During the active movements, the patient claims pain, and ROM reduction in flexion, extension and rotation movements is also observed. The repeated movements test shows directional preference towards extension. The Aberrant Movement test (Painful arc with flexion - Painful arc with return - Instability catch - Reversal of lumbo-pelvic rhythm - Gowers' sign) is positive due to the painful arc with flexion and painful arc with return. The maintained seated position reproduces usual pain.

Clinical conclusion

Non-specific LBP with directional preference towards extension and reduced lumbar stability. No relevant yellow flags or suspicion of red flags.

Treatment

This patient should be treated by following the usual musculoskeletal rehabilitation. The aims of the treatment are information concerning lumbar function, education regarding the seated position, and self-management through home treatment. Particular attention should be given to flexion intolerance, by using exercises aimed at progressive recovery of flexion and education on the better positions for sexual intercourse. Advice to stay active, manual therapy and repeated exercises based on directional preference should be later substituted with specific graded activity exercises and endurance exercises at the same time. Functional training addressed to sexual activity should complete this program.

- **Clinical case #2 – “PIPT” pathway**

History

A 30-year-old man reports an eight-month history of LBP located in the back central area and referred to gluteal/hip areas bilaterally.

This pain has progressively worsened over time, but it remains intermittent and mechanical in nature. His symptoms worsen in quick dynamic activities, like gym or during sexual activity (spontaneous confidence of the patient).

Furthermore, the patient reports loss of balance, fear of movements, and fear of getting hurt. There is a significant disability in ADLs and some activities are possible only by performing them slowly. The disability pattern also includes sexual disability: the patient reports decreased sexual activity caused by pain (“…without pain I could do sex more often…”) and fear of get worse the relationship with his partner (“…I'm afraid that my fiancée will get tired of me because we have less sex…).

Outcome Measures

NRS = 6 (acute, stabbing pain); ODI = 32 % (Item 8: My sex life is severely restricted by pain); PSEQ = 22/60; PCS = 32/52; TSK = 34/52.

Physical Exam

The patient’s posture appears rigid with guarding movements. The Aberrant Movement test is negative when performed slowly and positive when performed quickly. Trendelenburg and Active Straight Leg Raising tests are positive. Other movement tests like dead bug exercise or supine lower trunk rotation are performed with relevant apprehension.

Clinical conclusion

Motor control dysfunction in a patient with moderate disability during the ADLs and presence of yellow flags (fear of movement, fear-avoidance, and fear of re-injury).

Treatment

The treatment follows the PIPT model. The aims are information on pain, education and movements reconditioning, to stimulate pain self-efficacy and self-management. Graded activity exercises are proposed to improve abilities, safe feeling, and functional movements. In addition, suggestions concerning sexual positions and sexual activity are included. For example, because fast lumbo-pelvic movements cause pain due to poor motor control, the patient may initially modify sexual behavior by seeking pleasure through slower but more intense movements, or adopt positions that require less speed and less pelvic movement. It is mandatory treating and monitoring symptoms, discussing with the patient the therapeutic strategy, reassuring him, and documenting his progress by re-submitting questionnaires and clinical tests on time.

- **Clinical case #3 - "PIPT with referral" pathway**

History

A 52 year old woman, during a physical therapy session for LBP, speaks on the help her husband gives her in managing the house in relation to back pain, how she does shopping or housework, etc. Sarcastically smiling, she also says, "I no longer feel any desire for my husband, and I do it (meaning sex) just to make him happy".

Outcome Measures

ODI = 30 % (Item 8: My sex life is normal and causes no extra pain); HADS = 11; FSFI = 24; DAS = 40.

Assessment

Through a deepening of the anamnesis, the patient is asked if the amount and quality of sexual intercourse is related to LBP, and more specifically if the pain is present or worsens during or after sexual intercourse (e.g. during a movement, maintaining or changing position), if pain prevents intercourse, and if so how long it has been happening.

The patient reports that her problem is not pain, but for about a year, she has perceived less sexual desire for her partner, because she feels less pleasure in the genital area. She also reports that her gynecologist offered her hormone replacement therapy and a pelvic floor PT later consulted did not help her.

Clinical conclusion

The decrease in desire is not related to LBP, but to a possible gynecological cause.

Treatment

Support the patient in starting the prescribed treatments. Point out that, in case of little benefit, she could also consult a sexologist.

- **Clinical case #4 – "Immediate referral" pathway**

  History

  A 31 years-old woman is referred for LBP. During history, the PT asks a question about what could worsen her pain, and the patient replies: "It is my period". She has also felt a lot of pain in the abdomen for about two years and this kind of pain is getting worse. Furthermore, over the past five months, she started to feel this pain also at other times than during menstruation, and pain during intercourse has increased.

  Outcome Measures

  ODI = 41 % (Item 8: My sex life is severely restricted by pain); FSFI = 19.

  Assessment

  The patient is asked if she visited a gynecologist, and she answers positively, but the prescribed drugs seem not effective.

  Clinical conclusion

  This patient could have a gynecological pathology provoking pain.

  Treatment

  Advice to visit a pain specialized gynecologist is given to the patient.
